# “An odyssey without receiving proper care” – experts’ views on palliative care provision for patients with migration background in Germany

**DOI:** 10.1186/s12904-019-0392-y

**Published:** 2019-01-21

**Authors:** Maximiliane Jansky, Sonja Owusu-Boakye, Friedemann Nauck

**Affiliations:** Clinic for Palliative Medicine, University Medical Center Goettingen, Robert Koch-Str. 40, 37075 Goettingen, Germany

**Keywords:** Minority patients, Health care system, Expert interviews, Migrant patients, Palliative care

## Abstract

**Background:**

Migrants seem to be underrepresented in palliative care in Germany. Access barriers and challenges in care remain unclear. We aimed to provide a comprehensive insight into palliative care for migrants, using expert interviews.

**Methods:**

Interviews with experts on palliative and general health care for migrants were audiotaped and transcribed. Data analysis followed a qualitative content analysis method for expert interviews proposed by Meuser and Nagel.

**Results:**

In total, 13 experts from various fields (palliative and hospice care, other care, research and training) were interviewed. Experts identified access barriers on the health care system and the patient level as well as the sociopolitical level. Services don’t address migrants, who may use parallel structures. Patients may distrust the health care system, be oriented towards their home country and expect the family to care for them. In care, poor adaptation and inflexibility of health care services regarding needs of migrant patients because of scarce resources, patients’ preferences which may contradict professionals' values, and communication both on the verbal and nonverbal level were identified as the main challenges. Conflicts between patients, families and professionals are at risk to be interpreted exclusively as cultural conflicts. Palliative care providers should use skilled interpreters instead of family interpreters or unskilled staff members, and focus on training cultural competence. Furthermore, intercultural teams could enhance palliative care provision for migrants.

**Conclusions:**

Though needs and wishes of migrant patients are often found to be similar to those of non-migrant patients, there are migration-specific aspects that can influence care provision at the end of life. Migration should be regarded as a biographical experience that has a severe and ongoing impact on the life of an individual and their family. Language barriers have to be considered, especially regarding patients' right to informed decision making. The reimbursement of interpreters in health care remains an open question.

**Electronic supplementary material:**

The online version of this article (10.1186/s12904-019-0392-y) contains supplementary material, which is available to authorized users.

## Background

In 2017, 23.6% of the population in Germany had a migration background (i.e. persons themselves or one of their parents are not German citizens by birth [1], hereafter referred to as migrants, which may or may not include the person’s own migration), the largest group being people with Turkish background (14%), followed by Polish (11%) and Russian background (7%). Although migrants are of younger mean age than the overall population (35.4 vs 46.7 years), the mean duration of stay in Germany for all migrants was 20.5 years [1]. A considerable number especially from Turkey, Italy, Spain and former Yugoslavia are work migrants who migrated in the 1960s and have now reached the age of 70 [2]. Therefore, an increasing percentage of migrants can be expected to need palliative care. Migrants in Germany experience barriers to health care services in general [3, 4], and international research suggests that this may also be the case for specialized palliative care services [5–8]. In Germany, specialized palliative care is provided by specially trained physicians, nurses and other professions in interdisciplinary teams in a variety of institutions (palliative care units in hospitals, palliative home care teams, hospices). Because of patient’s severe and complex symptom burdens, access barriers and inadequate care may have severe consequences for patients and families [9]. Appropriate and early integration of specialized palliative care, however, can improve patients’ quality of life and symptom burden, and support informal caregivers [7, 8]. Studies from the US, UK and the Netherlands identified barriers that may limit migrant patients´ access to specialized palliative care, including insufficient language knowledge, prejudices against palliative care, lack of cultural competence of health care professionals, and services that are not adapted to their needs [6–8, 10, 11]. As international studies from the US and UK focus mainly on specific groups of patients, e.g. Afro-American, of Hispanic or South-Asian origin, their results may give hints for building hypotheses about access to and use of health care services, given that the migrant population in Germany is not comparable to these countries. A series of studies from the Netherlands by de Graaff and colleagues focused on palliative home care for migrants with Turkish and Moroccan background, who came to the Netherlands as laborers [7, 12–14]. This patient group is comparable to migrants with Turkish background, the largest migrant group in Germany, De Graaff and colleagues explored the perspectives of patients, relatives and health professionals, and also analyzed cases (patient, relative and care provider). They found that while some families preferred to care for the patient without professional help, others would have benefited from it, but didn’t use it because they lacked information [12]. Barriers to home care were understanding of illness and prognosis, family structures and decision-making-processes, living environment and lack of formal information and referral. They also found that patients and relatives view of what constitutes “good care” (e.g. maximum curative treatment until the end of life) may contradict the norms underlying palliative care (e.g. a focus on improving quality of life and on advance care planning) [13] In Germany, empirical data on migrants access to specialized palliative care as well as care-related problems is sparse [15–17], despite a rapid development of specialized palliative care structures [18]. An epidemiological study indicates that migrants may be underrepresented in specialized palliative care in Berlin [19]. The exploratory pilot study presented here consisted of two parts: an online survey of hospice and palliative care professionals experiences with palliative care for migrants with Turkish or Arabic background in Germany (results are published elsewhere [16]), and expert interviews on palliative care provision for migrants to gain a comprehensive insight into this mostly unknown.topic [20].

### Aim

We aim to transfer expertise in different fields of health care for migrants to specialized palliative care, and thereby identify possible access barriers to specialized palliative care, problems and challenges in care, and helpful resources regarding care for terminally ill migrants.

### Methods

#### Expert identification

Experts can be defined as people who are responsible for development, implementation or control of solutions, strategies or policies. They usually have a privileged access to knowledge about groups of persons or decision processes, and have a high level of aggregated and specific knowledge, but also procedural, non-explicit knowledge that is otherwise difficult to access [21].

We followed a theoretical sampling strategy. Data was collected until no new aspects came up during interviews. We identified experts using different methods: Scientific and grey literature and conference proceedings were searched for experts on health care for migrants in Germany. Institutions specialized in health care for migrants (e.g. ethno-medical center in Hannover, Malteser migrant medicine) were asked to name experts. Experts who were interviewed were asked to identify other experts which were then approached for interviews. All university chairs for palliative medicine were contacted and asked to identify experts. As examples for cities with a high rate of migrants, we conducted in-depth-searches (e.g. migrant organisations; city council etc.) in Berlin and Munich. In Berlin we also invited all palliative care services via email to signpost experts among their staff, or in institutions they worked with.

We initially planned to interview experts on specialized palliative care provision for migrants, but during the research process it became apparent that there were only very few experts on this topic in specialized palliative care (see Table 1). We therefore included experts from other, adjacent fields of health care practice, research, and training (e.g. transcultural psychiatry, research on transcultural issues or public health, transcultural nursing services, training of health professionals in transcultural competency), aiming to transfer their expertise on health care for migrants to specialized palliative care. All fields of expertise are listed in Table 1.

**Table 1:** Participants (*n* = 13)

Gender	Female	7
	Male	6
Migration background	Yes	6
	No	7
Field of expertise	Palliative care, physician (Adults)	1
	Palliative care, nurse (children)	1
	Outpatient and inpatient hospice care	2
	Diversity management in institutions for old people	1
	Diversity management in hospital	1
	Transcultural psychiatry	1
	Outpatient nursing care	1
	Training of transcultural competence	1
	Research (migration, diversity, religion and health)	4

#### Data collection

Data was assessed using semi-structured guided interviews as recommended for expert interviews [21]. The interview guide was constructed by MJ and SOB based on the literature, key topics were access to (palliative) care, difficulties in care and strategies to overcome problems with respect to migrant patients. It can be accessed as Additional file 1. The interviews were mostly conducted face-to-face, three were conducted via phone. Interviews lasted between 26 and 104 min. After obtaining written informed consent, interviews were audiotaped and transcribed verbatim. All interviews were conducted by MJ, who had worked in empirical research in palliative medicine for three years prior to the study. Because of her theoretical knowledge and practical experiences in the field, the interviewer acted as a quasi-expert in the interviews [21, 22].

#### Data analysis

The data analysis was conducted according to the qualitative content analysis method developed for expert interviews by Meuser and Nagel [21]. In this method, 1) interviews are paraphrased in sequences; 2) paraphrased sequences are then given one or more thematic codes, and 3) are thematically grouped within one interview. 4) A main headline is developed for each group of sequences. 5) Paraphrases are then thematically compared to identify common themes and arguments, which are again grouped. 6) Those themes are then categorized and described using abstract or theoretical concepts. Data was first coded by MJ and subsequently discussed with another research team member working in the field (SOB). Data analysis was conducted using the qualitative analysis software MaxQDA11.

## Results

### Participants

Between 03/2013 and 03/2014, 13 experts from health care, research, training and diversity management were interviewed. About half of the participants were female, six had a migration background themselves. For demographic data and fields of expertise, see Table 1. After 13 interviews, no new concepts arose during the interviews, and data collection was closed.

### Categories and themes

**Fig. 1:**
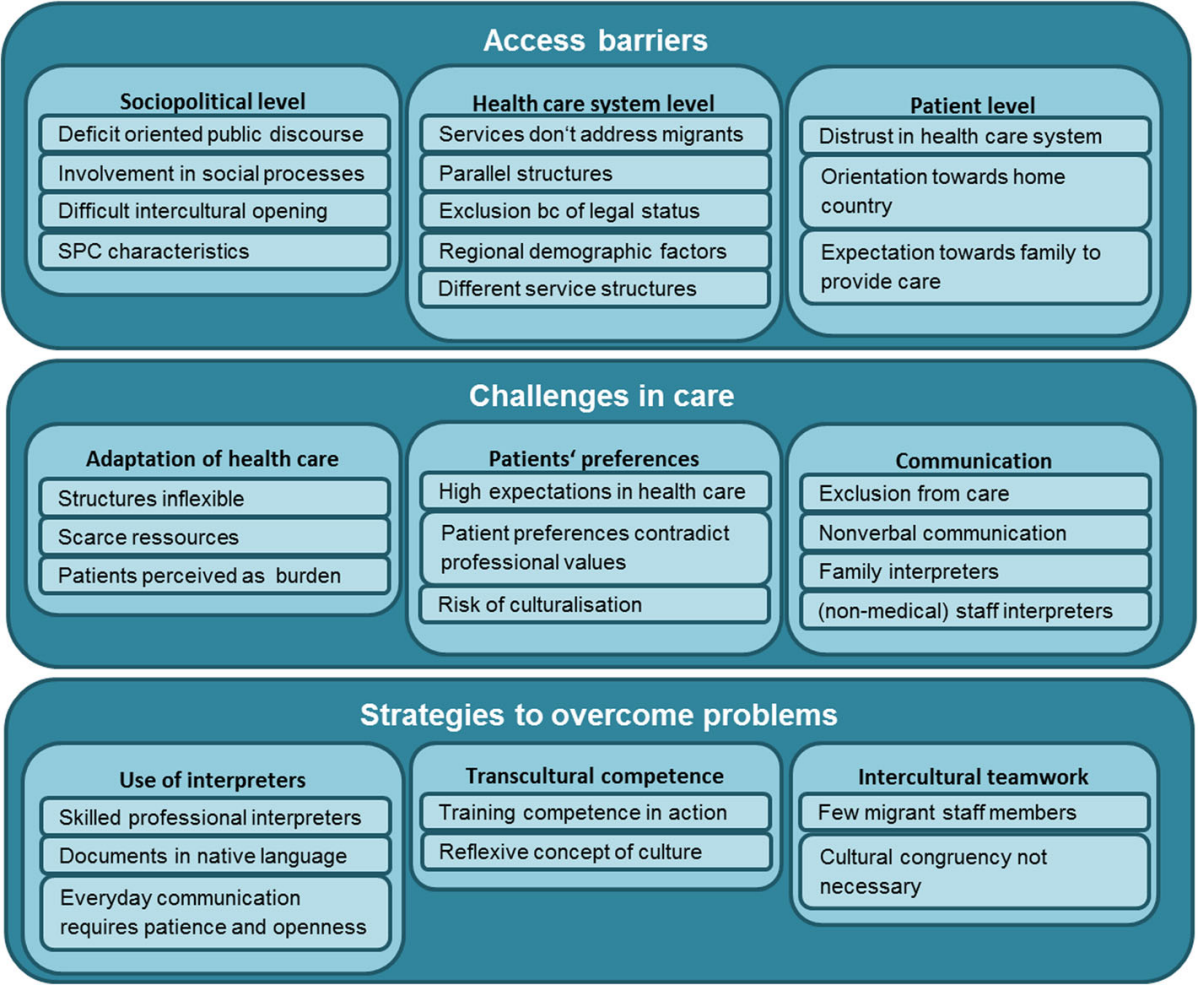
Categories and themes emerging in expert interviews

The following categories and underlying themes emerging from the expert interviews are presented structured by the interview guide’s key topics: *access to specialized palliative care*, *challenges in care*, and *strategies to overcome problems*. Figure 1 shows all categories and themes.

### Access barriers to palliative care

Access barriers to specialized palliative care emerged in the interviews in three categories: the *health care system*, concerning structures of health care institutions, and the migrant population, i.e. the *users.* A third category were *socio-political aspects* concerning the German society and its public discourse regarding both migration and palliative care

#### Health care system

Health care services mostly don’t explicitly address patients with migration background. Health and social services specifically addressing a migrant population, which were developed parallel to the health care system, hindered migrant integration into general health care. Some groups, e.g. undocumented immigrants; refugees who are only entitled to acute care, are at least partially systematically excluded from health care.

*“(That they) don’t get to the care structures they need, because information happened with an attitude of exclusion rather than inclusion (…) implicitly suggesting: ‘Better not come to us’, and the group of older immigrants just didn’t know what they needed, where to go (…) in this tangled mess of offers.” (Interviewee 3, health care professional, non-palliative care)*

Access may depend on regional demographics (high rate of migrant population), which may influence how important the development of strategies is perceived. Also, local service organization may influence access. An integration of specialized palliative care into curative care, e.g. may ease transitions to palliative care structures.

#### Users

Many migrants lack information about the health care system, and have problems navigating it. Some migrant patients may distrust the health care system due to previous experiences such as discrimination. They may be oriented towards their country of origin and its health care system, where often no palliative and/or hospice care exists.

*“Taboos are strengthened because the older generation longs for the younger ones to care for them at the end of life. But they can’t, and it’s a taboo to talk about this. Because what they think about nursing homes and end of life-care is shaped by institutional realities in the country of origin, and those are mostly bad examples.” (Interviewee 6, migration research)*

Furthermore, after receiving the diagnosis of a serious and incurable illness, they may expect families to care for sick family members.

“*(…) for those affected, this ‚nothing can be done‘* (in terms of curative options, AN) *is a death sentence, and they try to dive back into the old system of family and extended family support. (…) Then the memory of collective identity regarding social values subconsciously kicks in (…) that the family has to step in, even though they are all exorbitantly overburdened (…).” (Interviewee 7, health care professional, non-palliative care)*

#### Socio-political aspects

Experts also mentioned more general, sociopolitical aspects, which may impact migrants’ access to (palliative) care. Germany’s public discourse about immigration and migrants was perceived as deficit-oriented and negative. Migrants have not been appropriately involved in political and social processes, including changing paradigms of medical decision making from paternalism to shared decision making. They may therefore not be familiar with modern medical decision making e.g. the shift from protecting the patient from “harmful” information to full disclosure of diagnosis and prognosis, and involvement of the patient in all therapeutic decisions. The lack of societal integration may also strengthen a wish to someday return to their country of origin, especially for older migrants.

*“(…) it is like an encounter with biomedicine from the 1960s. And if you look around, how much has not been communicated, how much effort oncology has put into ending this pseudo-protection of patients, how much has been worked on shared-decision-making, work with the relatives in the past 30 years, that has all passed those people* (older migrants, AN) *by.” (Interviewee 7, health care professional, non-palliative care)*

Institutions or individuals in institutions that aim to strengthen diversity perceive these processes as laborious and time consuming. Also, specialized palliative care itself has only developed in recent years, and experts stated low awareness of specialized palliative care in the general public, which extends to the migrant population, and may be intensified by language barriers.

### Challenges in care

In the interview sections exploring challenges in care, three categories emerged: 1) Adaptation of health care structures to patients’ needs, 2) patients’ preferences, and 3) communication.

#### Adaptation of health care structures to patients’ needs

According to experts, health care structures may not be adequately adapted to the needs of migrant patients, and may lack the flexibility to adapt. Scarce resources, esp. time, and a lack of institutionalized strategies may result in health care providers being unable to cope with difficult situations, and therefore perceiving the migrant patient as a burden. This may also aggravate patients’ access barriers to proper care.

*“What we often experience is that patients who are labelled difficult, and this is often migrants, because of language barriers, because of cultural differences, because of different expectations towards the hospital, that they are sent from A to B, and that they often have experienced an odyssey without receiving proper care. And if they knew that there is another possibility of care, I believe this would be much more comfortable for the patients, and they would come to us sooner” (Interviewee 2, health care professional, palliative care)*

#### Patients’ preferences and risk of culturalisation

Patients’ care needs and wishes at the end of life are seen as similar regardless of migrant status. They wish for competent medical care and for professionals who dedicate their time to assess and fulfil their needs. Still, their preferences regarding interaction with health care professionals may be different. Patients may expect their physicians to decide for them, contrary to the current paradigm of shared decision making between patients and physicians.

*“My experience is that patients, especially from the Middle East, that they very often want a strict directive from their physicians with clear statements: ‘This is the situation, and this is how it is going to be.’ That is contrary to how we prefer to deal with (…) patients.” (Interviewee 2, health care professional, palliative care)*

At the same time, patients may prefer to not be fully informed about the disease, prognosis or treatment, and rather have the information given to a relative. This preference has to be respected and should be carefully documented. To be open to preferences and wishes of each patient was seen as paramount to good specialized palliative care; and experts highlighted the individuality of each patient.

*“(…) it is hard to accept that the patient is shielded from information, (…) we’re so used to holding up patient autonomy (…). I sometimes have problems to realize this myself, because it is the patient I treat. I have taken to ask the patient in private, if possible: (…) is it okay with you that your relative makes decisions for you? Or do you want to be involved in everything? (…) I document that, and then I can be sure it is okay with the patient.” (Interviewee 2, health care professional, palliative care)*

While experts mentioned possible differences in patient preferences, they also noted that migrant groups are as heterogeneous as non-migrants. They observed that issues in care for migrants were often attributed primarily to patients’ culture, and other factors (e.g. age, education, societal factors as discussed above) were not considered, leading to a risk to culturalize conflicts.

#### Communication and language barriers

Experts observed that language barriers hinder information of the patient and family about diagnosis, prognosis, and therapy options, and may subsequently lead to an exclusion of patients from optimum care. Language barriers may also extent to body language and non-verbal communication. Patients that are labeled as “not speaking German” may not be spoken to at all.

“*I (…) had a patient (…) with a brain tumor, who felt very isolated in medical care (…), not regarding the medical situation, but in a psychosocial manner. She had plenty of visitors. (…) but on the ward, they didn’t engage with her enough. (…) because they knew she didn’t speak German, they just put her food there, (…), and she would have wished they spoke to her. When there’s a note saying “doesn’t speak German” in the patient record, communication on our behalf may be terminated, and sometimes even non-verbal communication will be discontinued (…)” (Interviewee 7, health care professional, non-palliative care)*

Health care professionals often use family members as interpreters, and may lack awareness that this can be problematic, especially when dealing with difficult, intimate or burdening situations often occurring in specialized palliative care.

*“They* (health care professionals, AN) *should ask themselves, could they tell their father how the situation is, could they [tell] their spouse, when they only comprehend half of the words spoken, because they are in grief, if they could do so.” (Interviewee 8, health care professional, palliative care)*

Health care institutions often employ (non-medical) staff members as interpreters. These interpreter services may overburden staff members regarding both work load and emotion.

### Strategies to overcome problems

#### Overcoming access barriers

Specialized palliative care services should actively seek networks with migrant communities and services specialized on migrants. Experts stressed the importance of multipliers, who are rooted in migrant communities and can effectively disseminate information about palliative care.

*“I think it is easier to win over people from the community, who speak the language and to train them as multipliers (…) as intermediaries with regard to hospice and palliative care. To try to win people from the communities, and give them information they can disseminate” (Interviewee 9, migration research)*

Also, updating information on service provision regarding language as well as appropriateness, and disseminating information on specialized palliative care through appropriate channels (e.g. media in native language) were deemed helpful.

#### Use of interpreters

Good communication was seen as the basis of good care, and using skilled professional interpreters was recommended by experts. This requires planned and structured appointments with patients and families. In the experts’ opinions, using skilled interpreters for important conversations can facilitate communication in everyday care, will show appreciation of patients’ perspectives and may help them to feel better prepared to deal with the illness. Interpreter services should be offered by external, interculturally and medically educated professionals. The use of documents such as advance directives in native languages was seen as very important because of their legal consequences.

Some experts also mentioned that communication in everyday care was often possible without the use of interpreters by being open and patient, if care was more focused on the patient-professional-relationship.

*“(...) I get calls, and they say: ‚Do you have someone in your team who speaks, for example, Turkish? It’s very important, because it’s not possible to speak with the patient.’ Then I come and realize: It is possible to speak with the patient, you need to speak slowly and clearly, and take your time, and I think a lot of services don’t have that time (…)” (Interviewee 4, health care professional, palliative care).*

#### Training cultural competence

Experts described training of professionals’ cultural competence as an important premise for appropriate care. While many health care professionals express interest in transcultural training, only few institutions offer opportunities for training, and actively engage in training their staff. Those that participate in trainings often expect clear instructions and a toolkit to deal with migrant patients.

*“I don’t give them recipes – this is how you behave with someone from Turkey or from Afghanistan – but to approach them [patients, AN] with an open mind. (..) to tell them, when you’re insecure, or when you don’t understand something, to ask them. And also to explain yourself, what you’re doing and why.” (Interviewee 4, health care professional, palliative care).*

Experts on the other hand stressed the importance of training competence in action, e.g. by role play of uncomfortable situations, to reduce reservations and fear of breaking perceived taboos. The concept underlying those trainings should be “transcultural”, i.e. a reflexive concept of culture which takes socio-economical, gender and political factors into account.

#### Intercultural teamwork

There are only few migrant staff members in specialized palliative care. This may be due to a lack of formal qualification of migrant nurses. Migrant staff members may help to understand migrant patients and foster trust of patients and family. Nevertheless, all staff members should care for all patients, and cultural congruency between patient and professional was not seen as desirable.

*“On one hand, diversity management has to be implemented and practiced, and on the other hand, key positions should not only be filled with Germans.” (Interviewee 12, health care professional, non-palliative care)*

## Discussion

### Access barriers

The experts described many different possible access barriers to palliative care for migrant patients, on systemic and patient, but also societal level. Though immigration to Germany stretches far back, German politicians have only reluctantly declared it an immigration country, and the discussion about immigration has stressed negative issues, and demanded assimilation from migrants [23, 24]. Experts believe that this in turn lead to migrants being excluded from discourse in society, creating access barriers to (palliative) care. Lack of knowledge about palliative care was mentioned often for both migrant and non-migrant patients [17]. A representative survey showed that only 35% of the participating Germans could place the term “palliative” correctly [25]. Language barriers and lack of need-oriented information may exacerbate this problem. The proposed difference in access for different levels and settings of care, depending on the integration of palliative into curative care, e.g. oncology, is supported by the finding that migrant patients are less underrepresented in palliative care wards in hospitals than in outpatient settings or hospices [19].

Families may wish to care for patients at home despite needing professional support. Health care providers often assume that migrant families care for their terminally ill members and therefore don’t need as much professional support as non-migrant families [16]. Experts as well as other studies state that this is not regularly the case [7]. Low-threshold care offers (e.g. consultation and education about care) can help to ease transition into palliative care. Training and educating facilitators in the migrant communities about palliative care could improve access to specialized palliative care. In some regions, programs to improve the knowledge about the health care system in migrant communities already exist, and could be used to disseminate knowledge about palliative and hospice care.

### Patient preferences

Experts described that migrants may prefer a more passive interaction style, wanting the doctor to “tell them what to do”. This may be perceived as contradicting the paradigm of shared decision making between patients and physicians. This preference is difficult to separate from language barriers, which can be effective even if the patient apparently speaks German well [26]. In a review, Schouten and Meeuwesen found that ethnic minority patients were described as less verbally expressive and less assertive, which can also be due to the patient not fully understanding and being too embarrassed to ask questions [27].

Health professionals may find it difficult to balance giving realistic information about the prognosis and retaining patients’ hope, especially with patients they perceive as being “culturally different”. But realistic and hopeful information are not necessarily mutually exclusive. In a focus group study with patients and relatives with and without Muslim background, Oosterveld-Vlug and colleagues found that patients preferred realistic information regardless of their religious background [28]. This information should be provided in an empathic manner. Muslim participants stated that patients may prefer to have a relative informed first, and that information should be focused on the lack of curative options. Likewise, experts in our study stated that some patients wish to not be fully informed. Some patients may choose to give up part of their individual autonomy, and let others, family members or physicians, decide for them. Though this has been discussed in relation to patient’s cultural (especially Muslim) background [29–31], it is not limited to “non-Western” patient groups [32]. If patients express that they rather want information given to family members than to themselves, this should be respected, and patient wishes should be documented. Health care providers should also be aware that patients themselves may also feel that physicians are holding important information back or don’t try to overcome language barriers [26].

### Communication and language

Experts stressed the importance of good communication with migrant patients, and the benefit of using professionally trained and medically educated interpreters. Communication was also mentioned as a major issue by hospice and palliative care providers [16], general practitioners [7] and patients [18, 26]. Language barriers can hinder appropriate needs assessment [7] [12] and negatively affect the doctor/nurse-patient relationship [33] [34]. Health care providers should be aware how they communicate nonverbally, as nonverbal behavior of all parties involved must be interpreted within context [35]. In some situations, especially if care is focused on the relationship between patient and professional, an approach that involves patience, time and creativity may be helpful. Other situations that involve e.g. informing the patient about treatment options and discussing treatment goals, require a thorough reassurance of verbal understanding. Patients may not request more information when they don’t understand everything, because they feel embarrassed [26]. While some patients may prefer translation by a relative to a professional translator, this situation is often very burdening to the relative, especially regarding conversations around treatment goals and the transition from curative to palliative care [26, 36–39]. An international systematic review found that using professional interpreters in healthcare improved clinical care more than using ad-hoc interpreters like relatives or untrained staff members [40]. Despite the evidence supporting usefulness of professional interpreters, a recent report on cultural sensitivity of hospitals in the Germany found that 59% of hospitals never used professional interpreter services. Most hospitals (68%) often or always used patient’s relatives as interpreters [41]. At the moment, there are no regulations on the use of (professional) interpreters in health care settings in Germany. It also remains unclear which party is responsible for financing interpreter services. There may be regional initiatives offering interpretation in health care settings. Initiatives to implement coverage of interpreter services into social legislation have been unsuccessful. Especially in the light of the current integration of refugees, guidelines and clear instructions on the use of interpreters should be implemented on the national level. Baurer and colleagues state that professional interpreters are rarely used, even if they are available to health care professionals. They propose strategies to improve utilization, including training and educating clinicians on how to work with interpreters, and providing and promoting remote access to interpretation (e.g. via phone or video conference). Like the experts in our study, they recommend to train and educate bilingual staff so they can adequately interpret medical encounters [37]. These strategies should all be supported on an organizational level to be successfully implemented [42]. Not being able to understand makes patients especially vulnerable and dependent and ultimately restricts their right to make informed treatment decisions [26, 43]. Weber et al. have developed helpful guidelines for both health care institutions and individual health care professionals who want to implement and use interpreter services [42].

### Cultural competence

When experiencing conflicts with migrant patients, professionals may attribute those conflicts to cultural differences [44]. A training of scultural competence that focuses on communication processes instead of cultural differences may help to challenge stereotypes, focus on commonalities instead of differences, and subsequently improve care not only for migrant, but for all patients [45, 46]. Otherwise, a biographical sensitive approach with focus on individual biographical experiences can improve the patient-professional relationship, and understanding of patients’ needs and wishes, as suggested by Domening [45].

### Intercultural teamwork

Strengthening diversity within the palliative care team is an important factor for managing patient diversity [47]. Staff members with a different background can implement new perspectives and provide alternative interpretations for conflict situations.

### Limitations

Not all experts that were interviewed were closely familiar with the field of palliative care, only some had worked in clinical practice in palliative care. Therefore, important aspects that are unique to this field may have been missed. Despite this, the interviewed experts offered comprehensive and complex perspectives on different aspects of health care for migrants, and their experience from other fields of health care can enrich our knowledge about migrants in specialized palliative care. Some of the factors that influence access to and provision of care are specific for Germany (e.g. public discourse on migration). Nevertheless, most results, especially those on challenges in care and strategies to overcome problems are transferable to other countries and migrant or minority populations. Interviews took place before 2015, when Germany experienced a peak in refugee numbers. The specific situation of refugees was therefore not a focus in the interviews. This especially vulnerable group experiences specific access barriers to care due to legal status, and severe burden because of their psychosocial situation, e.g. family separation and living conditions [48–52].

## Conclusion

Though needs and wishes of migrant patients are found to often be similar to those of non-migrant patients [17, 53], there are migration-specific aspects that can influence care provision at the end of life. Migration should be regarded as a biographical experience that has a severe and ongoing impact on the life of an individual and their family [17, 54]. Legal aspects concerning migration status can influence access to and provision of care. Language barriers have to be considered, especially regarding patients’ right to informed decision making. In 2008, Babitsch et al. stated that to ensure culturally sensitive and competent health care, institutions have to consider and accept cultural diversity, and provide access to professional medical interpreters [33]. They also conclude that most German hospitals don’t fulfil these requirements, and it seems that little has changed in the last ten years. The reimbursement of interpreters in health care remains an open question. The use of professional medical interpreters, and intercultural awareness training for health professionals are widely recommended measures to ensure adequate health care for all patient groups [31, 33], and institutions providing hospice and palliative care are no exception.

Further research should focus on patient’s perspectives to deepen the understanding of their needs, and on care pathways for migrant patients to identify access barriers to appropriate end of life-care.

## Additional file

Additional file 1: Interview guide for expert interviews. (DOCX 17 kb)
