# Supplementary material for: “An odyssey without receiving proper care” – experts’ views on palliative care provision for patients with migration background in Germany
Source: BMC Palliat Care. 2019 Jan 21;18:8. doi: 10.1186/s12904-019-0392-y (PMC6341678; doi:10.1186/s12904-019-0392-y)
Supplement: Supplementary file 1 — Interview guide for expert interviews. (DOCX 17 kb) [file 12904_2019_392_MOESM1_ESM.docx]

**Interview guide for expert interviews**

- Introduction of the researcher and the research project.

*General questions*

- Please describe your work context.
- What experience do you have with health care for migrants?

*Access to care*

- How do migrant patients access health care? How do they access specialist care?
- *(For people working as health care providers)* How do migrants access your own care setting?
- Do you think that migrants use health care to the same extent as non-migrants?
- Are there access barriers?
  - What do you think causes them?
  - Can you describe a situation from your line of practice, where these access barriers occurred?
  - How could access be improved?

*Patient care*

- What are your experiences in practical health care for migrant patients?
- Are there specific challenges or problems?
- How are they dealt with?
  - Can you describe a situation from your line of practice, where these challenges occurred?

*Strategies to overcome problems*

- Have you or the institutions you work with developed strategies to overcome access barriers or challenges in care?
  - Can you describe a situation from your line of practice, where these strategies where used?
- Are these strategies used in practice? Are they experienced as helpful? Are there difficulties with implementation?
- If strategies are experienced as useful: Why do you think they work in your institution?
- If strategies are not used or not experienced as useful: Why do you think they are not used?
  - How could the use of these strategies be improved?

*Final question*

- If you imagine ideal conditions in which health care can be equally accessed by and is equally provided to migrant and non-migrant patients – what would these ideal conditions look like?
